# Knowing your neighbourhood: local ecology and personal experience predict neighbourhood perceptions in Belfast, Northern Ireland

**DOI:** 10.1098/rsos.160468

**Published:** 2016-12-07

**Authors:** James Gilbert, Caroline Uggla, Ruth Mace

**Affiliations:** 1 Department of Anthropology, University College London, 14 Taviton Street, London WC1H 0BW, UK; 2 Demography Unit, Department of Sociology, Stockholm University, 106 91 Stockholm, Sweden; 3 Life Sciences, Lanzhou University, 222 Tianshui South Road, Lanzhou, Gansu Province 73000, People's Republic of China

**Keywords:** life-history theory, ecological perceptions, mortality risk, morbidity risk

## Abstract

Evolutionary theory predicts that humans should adjust their life-history strategies in response to local ecological threats and opportunities in order to maximize their reproductive success. Cues representing threats to individuals' lives and health in modern, Western societies may come in the form of local ages at death, morbidity rate and crime rate in their local area, whereas the adult sex ratio represents a measure of the competition for reproductive partners. These characteristics are believed to have a strong influence over a wide range of behaviours, but whether they are accurately perceived has not been robustly tested. Here, we investigate whether perceptions of four neighbourhood characteristics are accurate across eight neighbourhoods in Belfast, Northern Ireland. We find that median age at death and morbidity rates are accurately perceived, whereas adult sex ratios and crime rates are not. We suggest that both neighbourhood characteristics and personal experiences contribute to the formation of perceptions. This should be considered by researchers looking for associations between area-level factors.

## 1. Background

Individuals have access to a limited amount of resources that they are able to invest in essential life processes such as survival and reproduction [1,2]. Trade-offs arise as individuals allocate time and energy units to specific functions, which cannot also be allocated elsewhere [3]. How successfully an individual navigates these trade-offs throughout its life course will contribute significantly to its reproductive success. Examples of classic life-history trade-offs include allocating resources between growth, maintenance or reproduction, whether to invest in current or future reproductive efforts, and whether to invest in few costly offspring or many less costly ones. Strategies can vary on whether they deliver rewards relatively soon or further into the future, with the most successful individuals employing strategies that are best adapted to their physical and social environments, as well as their internal developmental trajectory. Many life-history theorists propose that in harsh environments individuals should favour a fast life-history strategy with earlier maturation, earlier reproduction and higher levels of risk-taking [4,5]. Although a series of mathematical models recently suggested that the optimal response to variation in environmental harshness is not as simple as adopting a ‘faster’ or ‘slower’ strategy [6], it is clear that being able to adapt life-history behaviours appropriately to the local physical and social environment is fundamentally important to any organism's inclusive fitness.

In humans, there is growing evidence that characteristics of the local ecology correlate with various life-history outcomes. A classic study of the relationship between the conditions of 77 Chicago neighbourhoods and life-history behaviours found that areas with lower life expectancies had a higher proportion of teenage mothers [5]. The study also found that homicide rates and male life expectancy at birth, excluding the effects of local homicide rates, were highly correlated. The results suggest that life expectancy may be an important determinant of both the reproductive behaviour and violence-related risk-taking. Evidence has also been described of preventable male deaths, thought to be the outcome of risky behaviours, being positively associated with extrinsic mortality rates and crime rates at the area level in Northern Ireland [7]. This result is consistent with the life-history theory prediction that when unavoidable risks to an individual's survival are prevalent, the relative pay-off for investing in long-term strategies is lower and risky behaviours should be favoured. Extrinsic mortality and crime rates have been reported in association with a higher risk of early fatherhood in the same population, whereas a higher risk of early motherhood was found in areas of high extrinsic mortality rates, crime rates and female-biased sex ratios [8], suggesting that mate competition may be influencing fertility behaviour among females. A recent study of reproductive behaviour among eight Makushi communities in southern Guyana found that adult sex ratios (ASRs) were strongly associated with male mating effort [9]. In communities where males were in the minority, males were more willing to engage in uncommitted sexual activity but in areas with male-biased ASRs, males and females were equally willing to do so. The results suggest that when there is an abundance of males, it may be favourable for males with a partner to invest in their current relationship rather than a risky attempt to secure a second mate.

Natural selection may have favoured individuals who are able to make accurate inferences about their environment and adjust their behaviour accordingly. For example, an individual who is able to use the age profile of other individuals in the local population to infer age-specific mortality risk will be better able to adjust the timing of important life events compared with individuals who are less able to gauge such risks. Similarly, frequently encountering sick or unhealthy individuals might indicate that an individual's morbidity risk is high and that a fast life-history strategy should be favoured. While it is an implicit assumption of many life-history studies, whether individuals perceive their local environment accurately is largely an untested empirical question. Studies measuring perceptions have often focused on the overall perceived environmental quality [10], which does not aid our understanding of whether individuals can pick up particular aspects of their local ecology that are believed to influence life-history outcomes. Researchers' observations on the streets of two Newcastle neighbourhoods formed age profile estimations that did not map accurately on to census data for those neighbourhoods [11]. Perceptions of local ecologies, including life expectancy, ASRs and socioeconomic factors, have been found to predict self-reported attitudes towards violence and mating [12] in the same way that the actual neighbourhood characteristics predict violence and teen pregnancy rates at the area level in data from the UK [13]. These studies suggest that perceptions of local ecological characteristics are a means by which the environment can influence individual life-history strategies, but they did not test whether the perceptions were accurate.

To address this question, we collected perception data from residents of eight neighbourhoods in Belfast, Northern Ireland, where we have previously used Census data to test ecological effects on life-history outcomes [7,8]. We gathered individual perceptions of four neighbourhood characteristics, median age at death, morbidity rate, crime rate and ASR, and compared them with corresponding statistics provided by the Northern Ireland Statistics and Research Agency. If results show that individuals' perceptions of their local ecology map poorly to the real local conditions, then this might point to alternative mechanisms than psychological perceptions of an individual's local area for how human life-history strategies are formed.

## 2. Material and methods

### 2.1. Sample

We selected neighbourhoods that ranked in the top or bottom third for both a multiple deprivation measure, which included health deprivation and crime scores, and ASR, so that any effects were more likely to be captured. Neighbourhoods were defined using super output area (SOA) boundaries, which are local administrative units that have between 1300 and 2800 residents. Questionnaires were distributed door-to-door during May and June 2015 to residents of the eight neighbourhoods in Belfast, Northern Ireland (electronic supplementary material, S1). One hundred and fifty-nine individuals completed the questionnaire, which contained questions on individual demographic and socioeconomic information, and neighbourhood perceptions (electronic supplementary material, S2). Individuals who did not complete questions that formed variables used in the regression analyses were omitted from the relevant models.

### 2.2. Neighbourhood data

The neighbourhood statistics are explained in table 1. We used SOA-level data for all characteristics apart from median age at death, which was only available at the higher administrative level of the ward. We used the most recent datasets that were available at the time of the investigation, which were released between 2010 and 2014.

**Table 1.** Definition and sources of neighbourhood statistics.

neighbourhood characteristic	definition	source
median age at death	median age at death recorded in 2012	median age at death, Northern Ireland Statistics and Research Agency (NISRA) 2014 [14]
morbidity rate	percentage of residents with a ‘long-term health problem or disability: day-to-day activities limited a lot’	2011 Census, NISRA 2012 [15]
crime rate	crime ranks were based on rates of violence, robbery, public order, burglary, vehicle theft and criminal damage offences between 2004 and 2009	Northern Ireland multiple deprivation measure (NIMDM) report, NISRA 2010 [16]
adult sex ratio	the number of males divided by the number of females	2011 Census, NISRA 2012 [15]

### 2.3. Perception data

Using seven-point Likert's scale, respondents were asked to estimate the age at which most people in the neighbourhood lived to, to the nearest 5 year bracket (perceived median age at death); whether many people in the neighbourhood suffered from a long-term, limiting illness (perceived morbidity rate); how safe they felt in the neighbourhood (perceived personal safety); and whether there were more men or women in their neighbourhood (perceived ASR). As the ASR of an individual's own age group is likely to be the most relevant to the individual's life-history behaviours, we asked whether there were more men or women in ‘your age category’. Age categories, 18–40, 40–59 and 60 years or above, were described prior to the question being asked, and the census data used in analyses were the sex ratio of the individual's age category, within their particular neighbourhood.

### 2.4. Data analysis

General linear regressions were used to test how well individuals' perceptions mapped onto the actual characteristics of their neighbourhood. Each of the characteristics were tested in separate models controlling for age, sex, whether respondents left school before or after the age of 16 and household income (below £20 000, £20–40 000 and above £40 000). Regression coefficients (*B*), their standard errors (s.e. *B*), standardized regression coefficients (*β*) and *p*-values are reported. All analysis was performed in R Studio, v. 3.1.1 [17].

There is an ongoing debate in the literature regarding the analysis of ordinal data gathered from Likert-scale questions. Some have argued that non-parametric tests are more appropriate [18] but given the evidence and support for the use of parametric tests available in the literature [19,20], we felt that parametric general linear models were appropriate in this case. The respondents in this study were grouped by neighbourhood and a multilevel modelling approach would have been appropriate had we gathered data from towards 30 neighbourhoods, rather than the eight we sampled [21].

## 3. Results

**Figure 1.**
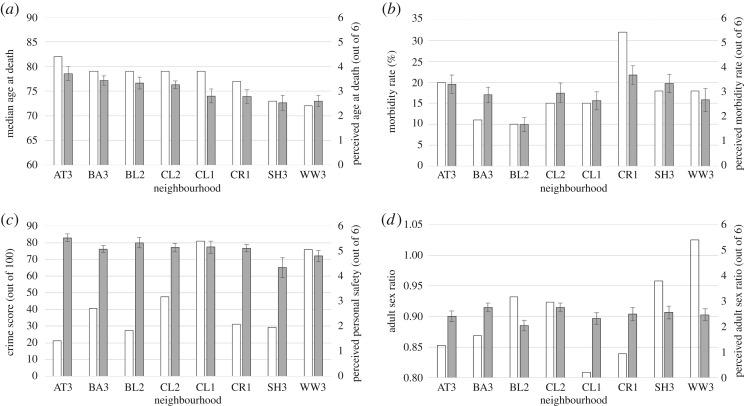
Neighbourhood characteristic and perceptions by neighbourhood. (*a*) Median age at death, (*b*) morbidity rate, (*c*) crime rate and perceived personal safety, (*d*) adult sex ratio. White bars , actual values; grey bars, perception scores.

The mean perceptions of each characteristic by neighbourhood are shown in figure 1*a*–*d* (correlations between individual perceptions of different characteristics available in electronic supplementary material, S4). In general, linear models controlling for sex, age, education and household income, perceptions of median age at death and morbidity rate were significantly predicted by the actual neighbourhood values (table 2). Mean responses for perceived median age at death were in the correct 5 year brackets for five out of the eight neighbourhoods, whereas three of the four neighbourhoods with the highest rates of morbidity had the three highest perception scores. Perceived ASR was not significantly predicted by actual ASRs, and neighbourhood crime scores did not predict perceived personal safety. Further analysis showed that personal exposure to vandalism and violence significantly predicted perceived personal safety controlling for demographic and socioeconomic variables (table 3 and electronic supplementary material, S3).

**Table 2.** Ability of neighbourhood characteristics to predict perceptions of (i) median age at death (MAD), (ii) morbidity rate, (iii) personal safety and (iv) adult sex ratio (ASR) in general linear models controlling for sex, age, age-squared, education and household income. *β,* standardized regression coefficient. *B* (s.e. *B*), regression coefficient (standard error). *n*, number of cases in each model after omitting respondents who did not answer all the relevant questions.

	(i) median age at death (*n* = 132)			(ii) morbidity rate (*n* = 133)		
	*β*	*B* (s.e. B)	*p*-value	*β*	*B* (s.e. *B*)	*p*-value
neighbourhood MAD (i)	0.19	0.07 (0.03)	0.04*	—	—	—
morbidity rate (ii)	—	—	—	0.29	0.26 (0.09)	0.004**
sex	0.01	0.02 (0.19)	0.93	0.02	0.06 (0.23)	0.84
age	−0.13	−0.01 (0.03)	0.79	1.01	0.10 (0.05)	0.04*
age-squared	0.32	0.00 (0.00)	0.51	−0.98	−0.00 (0.00)	0.05*
education post-16	0.03	0.07 (0.22)	0.76	0.06	0.21 (0.35)	0.55
income group 2	0.26	0.60 (0.22)	0.007**	0.08	0.28 (0.35)	0.42
income group 3	0.30	0.74 (0.27)	0.007**	0.06	0.21 (0.42)	0.61

	(iii) personal safety (*n* = 135)			(iv) adult sex ratio (*n* = 134)		
	*β*	*B* (s.e. *B*)	*p*-value	*β*	*B* (s.e. *B*)	*p*-value
neighbourhood crime (iii)	−0.01	−0.00 (0.00)	0.92	—	—	—
ASR (iv)	—	—	—	−0.03	−0.19 (0.65)	0.77
sex	−0.10	−0.20 (0.18)	0.26	−0.27	−0.51 (0.16)	0.002**
age	−0.05	−0.00 (0.03)	0.93	0.41	0.02 (0.03)	0.39
age-squared	0.17	0.00 (0.00)	0.74	−0.56	−0.00 (0.00)	0.25
education post-16	0.21	0.42 (0.20)	0.04*	−0.11	−0.21 (0.18)	0.24
income group 2	0.03	0.06 (0.21)	0.78	0.17	0.33 (0.19)	0.09
income group 3	0.02	0.05 (0.25)	0.84	0.27	0.57 (0.23)	0.01*

*p < 0.05; **p < 0.01.

**Table 3.** Ability of exposure to crime to predict perceived personal safety in separate general linear models controlling for sex, age, age-squared, education and household income. *β,* standardized regression coefficient. *B* (s.e. *B*), regression coefficient (standard error).

type of crime	*β*	*B* (s.e. *B*)	*p*-value
vandalism	−0.20	−0.38 (0.16)	0.02*
antisocial behaviour	−0.09	−0.15 (0.14)	0.29
violence	−0.22	−0.64 (0.25)	0.01*
sectarian threat/violence	−0.15	−0.36 (0.22)	0.10
street theft/burglary	0.12	−0.22 (0.16)	0.16

*p < 0.05

Of the individual characteristics, the age of respondents was positively associated with perceived neighbourhood morbidity (table 2). Respondents in the middle- and high-income groups estimated median ages of death to be higher than those in the lowest income group. Individuals who had been educated past the age of 16 felt safer in their neighbourhoods, and females perceived their neighbourhoods as more female-biased.

## 4. Discussion

We have tested the accuracy of individuals' ecological perceptions in an urban developed population. We found evidence that perceptions of local morbidity rates and median ages at death are fairly accurate, but no evidence that local crime rates and ASRs are accurately perceived. As far as we are aware, our study is the first to test perceptions of multiple neighbourhood characteristics over several different areas, while controlling for individual characteristics.

When asked questions regarding local mortality and morbidity, both of which were perceived accurately, many respondents referred to the age or health of individuals they knew personally, indicating that the age and health of proximate neighbours and family members might be salient cues when forming perceptions of age and morbidity profiles. This is particularly relevant given that some sufferers of chronic illnesses and particularly frail elderly people will be spending less time outside their homes, meaning their age and health status are less likely to be observed in the street. Researchers built age profiles based on estimations they made of the ages of individuals that they encountered in the streets of two Newcastle neighbourhoods. When they compared them with census data for the two areas, they found that the age profiles based on observations alone were not accurate [11]. As they were researchers, not residents of the neighbourhoods, they would not have received socially transmitted information about local residents which is a possible reason for why they did not form accurate age profiles in the areas they visited. Residents will also spend more time in their neighbourhoods than the researchers did for the study, and this could help more accurate perceptions to be formed. In our study, respondents of the middle- and high-income groups perceived higher mean ages at death than the low-income group. Given the well-documented link between socioeconomic position and health outcomes [22], the perceptions of individuals in higher income groups could reflect the ages of death of their high socioeconomic peers.

Interestingly, our results indicate that despite some areas having substantially higher crime rates than others, most respondents feel safe in their neighbourhoods; 80% of all respondents ‘agreed’ or ‘strongly agreed’ with the statement ‘I feel safe in this neighbourhood’ (electronic supplementary material, S2). Crime is unlikely to directly affect all the individuals of an area, and the generally high perceptions of personal safety may reflect a relatively low exposure to violent crime for most individuals. Further analysis showed that personal exposure to crime, particularly vandalism and violence, was a better predictor of perceived personal safety than neighbourhood crime rates (electronic supplementary material, S3). Bereavements of close family members or friends were found to be associated with a greater degree of future discounting, whereas overall exposure to the deaths of more distant acquaintances was not in a North American sample [23]. This suggests that being aware of deaths is not enough to affect future discounting but experiencing close bereavement is, in the same way that background crime rates were not associated with perceived personal safety but personal experience of crime was. Being educated beyond the age of 16 was associated with higher perceived personal safety, supporting previous findings that individuals of higher socioeconomic position feel safer and are less worried by crime [24]. The crime rates used in this study were based on a composite measure that omitted some violent crimes, notably rape, murder and attempted murder. We do not expect the inclusion of these crimes would change perceptions of personal safety, as violent and sexual crime at the ward-level is correlated very strongly with other forms of crime and the crime score used in the multiple deprivation measure across Northern Ireland (electronic supplementary material, S4).

Perceptions of ASRs did not appear to be accurate; we did not find any statistical association between perceived and actual ASRs for any age category. ASRs varied between neighbourhoods but this was not reflected in between-neighbourhood differences in perceptions. Overall, 51% of respondents thought their neighbourhoods had equal sex ratios, 22% thought it was slightly female-biased, 18% thought it was female-biased and 1% thought it was very female-biased. Only 8% of respondents thought there were more males than females in their neighbourhoods. Seven of our eight neighbourhoods were female-biased when combining residents of all ages, although four were male-biased among 18- to 39-year-olds, and three were male-biased among 40- to 59-year-olds (electronic supplementary material, S1). We asked participants to estimate the ASR of their neighbourhood for the age category to which they belonged, but it is possible they were unable to answer without being influenced by their perceptions of the total ASRs. Across all of Belfast, the sex ratio of 0.9 was female-biased (90 males for every 100 females), and this could also have influenced individual perceptions. Uggla & Mace [8] found evidence of a ward-level association between a female-biased sex ratio and earlier motherhood across Northern Ireland; it is possible that individuals in this population may be adjusting their behaviour in response to sex ratios at a subconscious level, despite the inaccurate perceptions reported here. The inaccurate perceptions may be due to small sample sizes of each age group in this study, or because behavioural adjustment may be more sensitive to the sex ratios of particular social and demographic groups if mating patterns are influenced by religious [25] or socioeconomic boundaries [26].

There are a number of other potential effects that could have influenced the results of this study. Respondents' perceptions of ages at death and morbidity rates could have been based on the socioeconomic position of the neighbourhoods, given the well-publicized association between socioeconomic conditions and health outcomes [27]. An assumption we make is that the neighbourhood-level is representative of an individual's local ecology, although this is not the only source of ecological information people are exposed to. In urban areas, people regularly travel outside of their neighbourhoods to areas in which they may be exposed to mating opportunities and competition, threat of crime and cues relating to mortality and morbidity risks. In a previous study, perceptions of trust and paranoia among residents were mirrored by the perceptions of researchers who had spent just 45 minutes in two UK neighbourhoods, demonstrating how quickly perceptions can come to reflect the areas they are formed in [28]. Further, all individuals in modern populations will consume different forms of media that might impact perceptions. It is not possible to say whether local neighbourhoods, other areas that individuals visit or cues received from external media sources are likely to be stronger influences of individual life-history strategies. In this study, we analysed between-neighbourhood variation in the accuracy of perceptions using estimations made in response to a questionnaire. Neighbourhood perceptions could be explored in future research through a number of other methods, such as through priming individuals in ways that may affect their beliefs regarding their neighbourhoods in order to test the robustness of existing perceptions.

## 5. Conclusion

There is increasing evidence that life-history strategies vary with local ecological conditions in developed populations, and that factors other than individual socioeconomic characteristics underpin such patterns. In this study, we found that only two out of the four neighbourhood characteristics were perceived accurately. The possibility that ecological perceptions might not be accurate has implications for theories assuming that life-history strategies respond to local conditions via perceptions and behavioural adjustments. For example, it has been suggested that responses to local ecologies can be internalized by affecting the individual's somatic state, rather than being driven through psychological perceptions [29], although these mechanisms are not mutually exclusive. We have discussed the large contribution of local conditions to the formation of perceptions and speculated on the potential influence of personal experiences. At present, the psychological processes underlying the perception and retrieval of ecological information are poorly understood. A challenge for future studies will be to improve our understanding of how perceptions are formed and in what ways they influence life-history strategies.

## Supplementary Material

Neighbourhood Statistics. Key statistics for each neighbourhood sampled

## Supplementary Material

Questionnaire. Questionnaire used to gather individual data

## Supplementary Material

Personal Experience of Crime. Regression models testing effect of personal exposure to crime on perceived personal safety

## Supplementary Material

Correlation of Crime. Correlation of violent and sexual crime with other types of crime and crime score at the ward-level

## Supplementary Material

Raw Data

## Data accessibility

All primary data and supplementary materials are available online at http://rsos.royalsocietypublishing.org/. Statistical analyses were carried out in R Studio, available at https://www.rstudio.com/.
