# Supplementary material for: Knowing your neighbourhood: local ecology and personal experience predict neighbourhood perceptions in Belfast, Northern Ireland
Source: R Soc Open Sci. 2016 Dec 7;3(12):160468. doi: 10.1098/rsos.160468 (PMC5210677; doi:10.1098/rsos.160468)
Supplement: Neighbourhood Statistics. Key statistics for each neighbourhood sampled [file rsos160468supp1.docx]

**ESM 1: Neighbourhood Information**

Neighbourhood statistics- deprivation (MDM) rank, median age at death (MAD), percentage of residents with a long-term limiting illness, crime score, adult sex ratio (ASR), adult sex ratio rank, percentage of Catholic and Protestant residents, total number of households and the percentage of households that completed a questionnaire.

| **SOA** | **MDM Rank** | **MAD** | **Morbidity Percentage** | **Crime score (/100)** | **ASR Rank** | **ASR** | **Percentage Catholic** | **Percentage Protestant** | **Total households** | **Percentage households** |
| --- | --- | --- | --- | --- | --- | --- | --- | --- | --- | --- |
| Andersonstown 3 | 74 | 82 | 20 | 21.3 | 39 | 0.88 | 93.8 | -- | 641 | 2.7 |
| Ballyhackamore 3 | 108 | 79 | 11 | 40.7 | 10 | 0.8 | 14.31 | 70.09 | 1005 | 2.4 |
| Bloomfield 2 | 117 | 79 | 10 | 27.5 | 141 | 1.17 | 13.69 | 74.27 | 701 | 3.4 |
| Cliftonville 2 | 91 | 79 | 15 | 47.7 | 142 | 1.21 | 77.3 | -- | 623 | 3.4 |
| Cliftonville 1 | 49 | 79 | 14 | 81 | 19.5 | 0.83 | 88.33 | 6.11 | 770 | 3.2 |
| Crumlin 1 | 18 | 77 | 32 | 31.3 | 45 | 0.89 | 6.29 | 87.82 | 1061 | 1.8 |
| Shaftesbury 3 | 30 | 73 | 18 | 29.3 | 124.5 | 1.06 | 13.85 | 71.87 | 1172 | 1.5 |
| Water Works 3 | 43 | 72 | 18 | 75.9 | 139 | 1.15 | 85.65 | 7.23 | 815 | 1.8 |

MDM= multiple deprivation measure (high ranked neighbourhoods are more deprived), MAD= median age at death. Morbidity percentage refers percentage of residents with “a long-term health problem or disability: day-to-day activities limited a lot”. Crime score out of 100: higher score means more crime. ASR= adult sex ratio, ‘total households’ refers to total number of households in the SOA, ‘percentage households’ refers to the percentage of households in each SOA in which someone completed a questionnaire. Central line divides affluent (top) and deprived neighbourhoods.

**Adult Sex Ratios by Age Group**

Adult Sex Ratios (ASR) of each neighbourhood and Belfast's sex ratio overall. ASRs split into age categories and given for all ages combined. ASR calculated by dividing the number of males by the number of females; an ASR greater than 1.00 indicates more males than females.

| **Neighbourhood** | **AT3** | **BA3** | **BL2** | **CL1** | **CL2** | **CR1** | **SH3** | **WW3** | **Belfast** |
| --- | --- | --- | --- | --- | --- | --- | --- | --- | --- |
| 18-39 ASR | 1.04 | 0.89 | 0.99 | 0.79 | 1.19 | 0.87 | 1.03 | 1.19 | 0.96 |
| 40-59 ASR | 0.79 | 0.97 | 1.01 | 0.86 | 0.89 | 0.96 | 1.10 | 1.13 | 0.95 |
| 60+ ASR | 0.75 | 0.72 | 0.76 | 0.77 | 0.71 | 0.74 | 0.69 | 0.64 | 0.73 |
| Total ASR | 0.85 | 0.87 | 0.93 | 0.81 | 0.92 | 0.84 | 0.96 | 1.03 | 0.90 |
