# Supplementary material for: Knowing your neighbourhood: local ecology and personal experience predict neighbourhood perceptions in Belfast, Northern Ireland
Source: R Soc Open Sci. 2016 Dec 7;3(12):160468. doi: 10.1098/rsos.160468 (PMC5210677; doi:10.1098/rsos.160468)
Supplement: Questionnaire. Questionnaire used to gather individual data [file rsos160468supp2.docx]

*Hi, my name is X, and I’m a student at University College London (show UCL ID). I’m doing a student research project on people’s attitude towards their health and neighbourhood here in Belfast and we would be very grateful if you would take a few minutes to answer some questions. Is that OK?*

*Are you a permanent resident at this address? If not, is someone who lives there permanently at home?* IF NO PERMANENT RESIDENTS- THANK FOR HELP AND LEAVE.

| 1. **What is your gender:** | - Male | - Female |
| --- | --- | --- |

1. **What is your year of birth? ________**
2. **What is the postcode of this address? ____________**
3. **How long have you lived in your neighbourhood?**

| - - - < 1 year | - - - 11 - 20 years |
| --- | --- |
| - - - 1 – 5 years     - 6 – 10 years | - - - >20 years     - Whole Life (GO TO Q7) |

1. **What area did you live in before moving to this one?** Postcode previous area: _____
2. **How long did you live there?** ____ years
3. **What is your country of birth?**

| - - - Northern Ireland     - Republic of Ireland     - Scotland | - - - England     - Wales     - Other ____________ |
| --- | --- |

1. **What is your current employment status? Tick all that apply**

| - - - Unemployed     - Employed full-time     - Employed part-time | - - - Student full-time     - Student part-time     - Retired |
| --- | --- |

1. **What is the highest educational level you have completed?**

| - - - Primary school     - GCSE / O-Level / NVQ Level 1-2 / BTEC Level 1-2 / Equivalent     - A-Level / NVQ Level 3 / BTEC Level 3 / Equivalent | - - - Undergraduate / Equivalent     - Graduate / Post-graduate / Equivalent     - Other: __________     - None |
| --- | --- |

1. **What is your religion?**

| - - - Catholic     - Church of Ireland     - Presbyterian     - Methodist | - - - Other Protestant religion ________     - Other religion _________     - Agnostic     - No religion |
| --- | --- |

1. **Which of these types of living arrangement best describes your situation?**

| - - - Living alone     - Living with partner only     - Living with children only     - Living with partner and children     - Living with parents | - - - Living with parents and with partner or children     - Shared accommodation     - Other: ____________ |
| --- | --- |

1. **What is your marital status:**

| - - - Single     - In a relationship     - Married | - - - Separated/divorced     - Widowed / Widower |
| --- | --- |

1. **How many children of your own do you have? ______**
2. **At what age did you have your first child? ______**
3. **Is that the child of your current partner? Yes No**
4. **Does your household own or rent this accommodation?**

| - - - Own     - Part own, part rent     - Rent: private | - - - Rent: room of shared house     - Rent: social housing     - Live rent free, relationship to owner:   ___________________________ |
| --- | --- |

1. **How many cars does your household have access to? 0 1 2+**
2. **What is the total household annual income (yourself, your partner and your parents if living with them) before tax?** (show them sheet, don’t ask out aloud)

| - - - £0 - £5,000     - £5,001 - £10,000     - £10,001 - £15,000     - £15,001 - £20,000     - £20,001 - £25,000     - £25,001 - £30,000 | - - - £30,001 - £40,000     - £40,001 - £50,000     - £50,001 - £75,000     - £75,001 - £100,000     - £100,001 - £200,000     - More than £200,000 |
| --- | --- |

1. **Does your (collective family) income cover your household costs?**

| No, costs are considerably higher | No, costs are slightly higher | Yes, income and  costs are similar | Yes, with a small surplus | Yes, comfortably |
| --- | --- | --- | --- | --- |

*I will now ask you some questions about your wellbeing and neighbourhood.*

1. **How confident are you that you'll live until you are:**

| 40 | Not at all  confident | Slightly  confident | Confident | Very  confident |
| --- | --- | --- | --- | --- |
| 50 | Not at all  confident | Slightly  confident | Confident | Very  confident |
| 60 | Not at all  confident | Slightly  confident | Confident | Very  confident |
| 70 | Not at all  confident | Slightly  confident | Confident | Very  confident |
| 80 | Not at all  confident | Slightly  confident | Confident | Very  confident |
| 90 | Not at all  confident | Slightly  confident | Confident | Very  Confident |

1. **How confident are you that you'll live *HEALTHILY* until you are:**

| 40 | Not at all  confident | Slightly  confident | Confident | Very  Confident |
| --- | --- | --- | --- | --- |
| 50 | Not at all  confident | Slightly  confident | Confident | Very  confident |
| 60 | Not at all  confident | Slightly  confident | Confident | Very  confident |
| 70 | Not at all  confident | Slightly  confident | Confident | Very  confident |
| 80 | Not at all  confident | Slightly  confident | Confident | Very  confident |
| 90 | Not at all  confident | Slightly  confident | Confident | Very  confident |

1. **Do you think you will die of old age? Yes No**
2. **Most people in this neighbourhood live until they are?**

| 60-64 | 65-69 | 70-74 | 75-79 | 80-84 | 85-89 | 90+ |
| --- | --- | --- | --- | --- | --- | --- |

**How much do you agree with the following statements?**

1. ***On a regular day, I come into contact with an equal number of men and women***

| Strongly disagree | Disagree | Somewhat Disagree | Neither agree nor disagree | Somewhat agree | Agree | Strongly agree |
| --- | --- | --- | --- | --- | --- | --- |

1. ***In your age category, are there more men or more women in this neighbourhood?***

Age category: 18-39 40-59 60+

| Many more women | More women | Slightly more women | Equal numbers of both | Slightly more men | More men | Many more men |
| --- | --- | --- | --- | --- | --- | --- |

1. ***Many people in this neighbourhood suffer from a long-term limiting illness***

| Strongly disagree | Disagree | Somewhat Disagree | Neither agree nor disagree | Somewhat agree | Agree | Strongly agree |
| --- | --- | --- | --- | --- | --- | --- |

1. ***Compared to the people in my neighbourhood, I feel I have:***

| Much less than most | Less than most | Slightly less than most | The same as most | Slightly more than most | More than most | Much more than most |
| --- | --- | --- | --- | --- | --- | --- |

1. ***I feel safe in this neighbourhood***

| Strongly disagree | Disagree | Somewhat Disagree | Neither agree nor disagree | Somewhat agree | Agree | Strongly agree |
| --- | --- | --- | --- | --- | --- | --- |

1. ***There are many people in my neighbourhood I consider to be my friends***

| Strongly disagree | Disagree | Somewhat Disagree | Neither agree nor disagree | Somewhat agree | Agree | Strongly agree |
| --- | --- | --- | --- | --- | --- | --- |

1. **Whilst living in this neighbourhood, how often have you experienced any of the following?**

|  | **Not often a problem** | **Sometimes a problem** | **Often a problem** | **Very often a problem** |
| --- | --- | --- | --- | --- |
| **Vandalism** |  |  |  |  |
| **Antisocial behaviour** |  |  |  |  |
| **Violence towards you** |  |  |  |  |
| **Sectarian threat/violence** |  |  |  |  |
| **Street theft/burglary** |  |  |  |  |

1. **In the past 12 months, have you experienced any of the following life events:**

| - - - Loss of family member or friend | - - - Self/family/friend diagnosed with serious illness |
| --- | --- |
| - - - Loss of job | - - - Marital problems |
| - - - Moved house | - - - Witnessed or been the victim of a crime |
| - - - Had a child |  |

1. **In what ways do you have contact with your local community? Please tick all that apply**

- I work within the neighbourhood
- I attend residents and/or council meetings
- I am active in local community groups (such as sports team, charity and church groups, etc)
- I go to church locally
- I go to my local community centre/pub
- I keep up to date with community news via my local paper
- My children go to the local school
- I am not involved much with my local community

*Now some questions about your health and lifestyle*

1. ***I feel I am able to influence my own health to a large extent***

| Strongly disagree | Disagree | Somewhat Disagree | Neither agree nor disagree | Somewhat agree | Agree | Strongly agree |
| --- | --- | --- | --- | --- | --- | --- |

1. ***I often find myself worrying about things that may or may not happen***

| Strongly disagree | Disagree | Somewhat Disagree | Neither agree nor disagree | Somewhat agree | Agree | Strongly agree |
| --- | --- | --- | --- | --- | --- | --- |

1. **In general would you say your health is:**

| - - - Excellent     - Good     - Fair | - - - Poor     - Very Poor |
| --- | --- |

1. **Do you have any long-term limiting illnesses? Yes No**
2. **How often do you take part in any of the following physical activities?**

| **Activity** | **Never** | **1-3 times a month** | **Once a week** | **In last week** |
| --- | --- | --- | --- | --- |
| **Swimming** |  |  |  |  |
| **Hiking** |  |  |  |  |
| **Competitive sport** |  |  |  |  |
| **Cycling** |  |  |  |  |
| **Yoga** |  |  |  |  |
| **Jogging/running** |  |  |  |  |
| **Walking (>10 mins)** |  |  |  |  |
| **Strenuous housework** |  |  |  |  |
| **Other, please specify:**  **-**  **-** |  |  |  |  |

1. **How many cigarettes have you smoked in the last 24 hours?**

| - - - 0     - 1-5     - 6-10 | - - - 11-20     - 20+ |
| --- | --- |

1. **Do you ever drink alcohol? Yes No**
2. **How many units of alcohol have you consumed in the last week?**

| **Drink** | **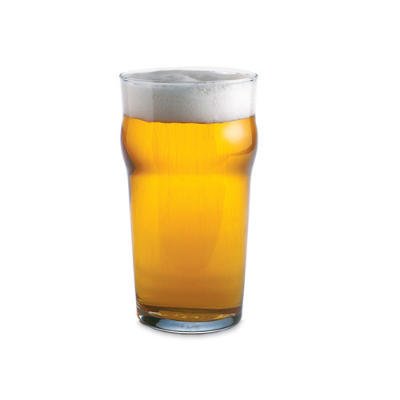**  **Pint**  **Lager/Cider/Ale**  **3-4%**  **2 units** | **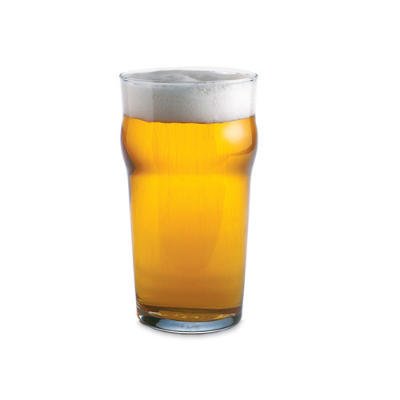**  **Pint**  **Lager/Cider/Ale**  **> 5%**  **3 units** | **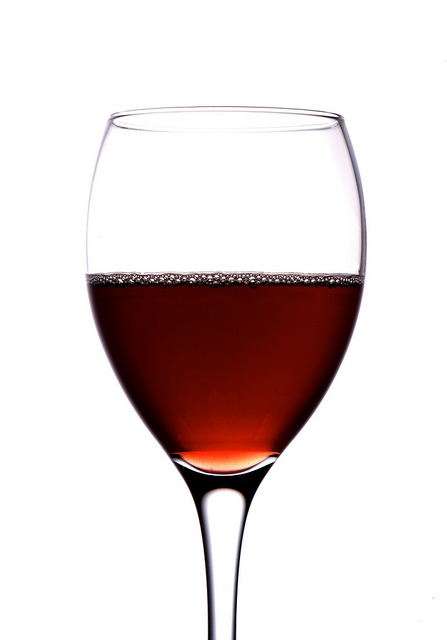**  **Glass 175ml**  **Wine**  **11-14%**  **2 units** | **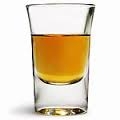**  **Single measure 25ml**  **Spirit**  **> 35%**  **1 unit** |
| --- | --- | --- | --- | --- |
| **Number consumed** |  |  |  |  |
| **Total Units** |  |  |  |  |

1. **On how many occasions have you consumed alcohol in the last week?**
2. ***I believe I do enough to look after my health***

| Strongly disagree | Disagree | Somewhat Disagree | Neither agree nor disagree | Somewhat agree | Agree | Strongly agree |
| --- | --- | --- | --- | --- | --- | --- |

*Thank you very much for taking part in this survey.*
