# Supplementary material for: Knowing your neighbourhood: local ecology and personal experience predict neighbourhood perceptions in Belfast, Northern Ireland
Source: R Soc Open Sci. 2016 Dec 7;3(12):160468. doi: 10.1098/rsos.160468 (PMC5210677; doi:10.1098/rsos.160468)
Supplement: Personal Experience of Crime. Regression models testing effect of personal exposure to crime on perceived personal safety [file rsos160468supp3.docx]

**ESM 3: Regression Models Testing Effect of Personal Exposure to Crime on Perceived Personal Safety**

We tested the effect of personal exposure to crime on perceived personal safety in general linear models controlling for sex, age, age-squared, educational level achieved (whether respondents left school at 16 or later) and household income (below £20,000, £20-40,000 and above £40,000). The tables below are the models testing the effect on perceived personal safety of the following crimes: vandalism, antisocial behaviour, violence, sectarian threat/violence, street theft/burglary.

|  | **Regression Coefficient** | **Std. Error** | **Standardized Regression Coefficient** | **p-value** |
| --- | --- | --- | --- | --- |
| Intercept | 4.97 | 0.67 | 0.00 | <0.01 |
| Vandalism | -0.38 | 0.16 | -0.20 | 0.02 |
| Sex | -0.20 | 0.17 | -0.10 | 0.24 |
| Age | -0.01 | 0.03 | -0.09 | 0.85 |
| Age-squared | 0.00 | 0.00 | 0.20 | 0.68 |
| Education post-16 | 0.46 | 0.20 | 0.23 | 0.02 |
| Income Group 2 | 0.01 | 0.20 | 0.01 | 0.94 |
| Income Group 3 | 0.01 | 0.25 | 0.003 | 0.98 |

|  | **Regression Coefficient** | **Std. Error** | **Standardized Regression Coefficient** | **p-value** |
| --- | --- | --- | --- | --- |
| Intercept | 4.87 | 0.69 | 0.00 | <0.01 |
| Antisocial Behaviour | -0.15 | 0.14 | -0.09 | 0.29 |
| Sex | -0.22 | 0.17 | -0.11 | 0.20 |
| Age | -0.00 | 0.03 | -0.02 | 0.96 |
| Age-squared | 0.00 | 0.0003 | 0.13 | 0.79 |
| Education post-16 | 0.42 | 0.20 | 0.21 | 0.04 |
| Income Group 2 | 0.01 | 0.21 | 0.01 | 0.95 |
| Income Group 3 | 0.03 | 0.25 | 0.01 | 0.91 |

|  | **Regression Coefficient** | **Std. Error** | **Standardized Regression Coefficient** | **p-value** |
| --- | --- | --- | --- | --- |
| Intercept | 4.91 | 0.67 | 0.00 | <0.01 |
| Violence | -0.64 | 0.25 | -0.22 | 0.01 |
| Sex | -0.26 | 0.17 | -0.13 | 0.13 |
| Age | -0.00 | 0.03 | -0.06 | 0.91 |
| Age-squared | 0.00 | 0.00 | 0.16 | 0.74 |
| Education post-16 | 0.41 | 0.20 | 0.21 | 0.04 |
| Income Group 2 | 0.07 | 0.20 | 0.03 | 0.75 |
| Income Group 3 | -0.01 | 0.25 | -0.01 | 0.96 |

|  | | **Regression Coefficient** | | **Std. Error** | | **Standardized Regression Coefficient** | | **p-value** | |
| --- | --- | --- | --- | --- | --- | --- | --- | --- | --- |
| Intercept | | 4.97 | | 0.69 | | 0.00 | | <0.01 | |
| Sectarian Threat/Violence | | -0.36 | | 0.22 | | -0.15 | | 0.10 | |
| Sex | | -0.24 | | 0.17 | | -0.12 | | 0.17 | |
| Age | | -0.01 | | 0.03 | | -0.10 | | 0.84 | |
| Age-squared | | 0.00 | | 0.00 | | 0.20 | | 0.69 | |
| Education post-16 | | 0.40 | | 0.20 | | 0.20 | | 0.05 | |
| Income Group 2 | | 0.03 | | 0.21 | | 0.01 | | 0.89 | |
| Income Group 3 | | 0.06 | | 0.25 | | 0.03 | | 0.82 | |
|  | **Regression Coefficient** | | **Std. Error** | | **Standardized Regression Coefficient** | | **p-value** | |  |
| Intercept | 4.80 | | 0.68 | | 0.00 | | <0.01 | |  |
| Street Theft/Burglary | -0.22 | | 0.16 | | -0.12 | | 0.16 | |  |
| Sex | -0.19 | | 0.17 | | -0.10 | | 0.28 | |  |
| Age | 0.00 | | 0.03 | | 0.02 | | 0.97 | |  |
| Age-squared | 0.00 | | 0.00 | | 0.11 | | 0.83 | |  |
| Education post-16 | 0.39 | | 0.20 | | 0.19 | | 0.055 | |  |
| Income Group 2 | 0.04 | | 0.21 | | 0.02 | | 0.86 | |  |
| Income Group 3 | 0.04 | | 0.25 | | 0.02 | | 0.87 | |  |
