# Supplementary material for: Knowing your neighbourhood: local ecology and personal experience predict neighbourhood perceptions in Belfast, Northern Ireland
Source: R Soc Open Sci. 2016 Dec 7;3(12):160468. doi: 10.1098/rsos.160468 (PMC5210677; doi:10.1098/rsos.160468)
Supplement: Correlation of Crime. Correlation of violent and sexual crime with other types of crime and crime score at the ward-level [file rsos160468supp4.docx]

**ESM 4: Correlation of violent and sexual crime with other types of crime and crime score at ward-level**

The crime rates used in this study were based on a composite measure including violence, robbery, public order, burglary, vehicle theft and criminal damage, but omitted more violent crimes, notably rape, murder and attempted murder. We do not expect the inclusion of these crimes would change perceptions of personal safety, as violent and sexual crime at the ward-level is correlated very strongly with other forms of crime and the crime score used in the multiple deprivation measure across Northern Ireland.

Correlations were tested between violent and sexual crimes and burglary, theft, criminal damage, drug-related crime, other crimes and the multiple deprivation crime and disorder score for each ward. Correlations reported for all wards of Northern Ireland (N=582), the neighbourhoods used in this study (n=7, two SOAs from same ward).

In the tables below, correlation coefficients are italicised and p-values are underlined. Values of zero indicate where R has returned a value of zero as the actual value was negligible. P<0.05 for all correlations tested.

| Ward-level crime correlation for all wards of Northern Ireland (N=582). Underlined: p-value. Italicised: Correlation coefficient | | | | | | |
| --- | --- | --- | --- | --- | --- | --- |
|  | Violence, Sexual Crime and Robbery | Burglary | Theft | Criminal Damage | Drug-related | Other Crime |
| Violence, Sexual Crime and Robbery | - | <0.01 | <0.01 | <0.01 | <0.01 | <0.01 |
| Burglary | *0.65* | - | <0.01 | <0.01 | <0.01 | <0.01 |
| Theft | *0.88* | *0.71* | - | <0.01 | <0.01 | <0.01 |
| Criminal Damage | *0.86* | *0.8* | *0.85* | - | <0.01 | <0.01 |
| Drug-related | *0.84* | *0.62* | *0.81* | *0.81* | - | <0.01 |
| Other Crime | *0.91* | *0.64* | *0.91* | *0.86* | *0.84* | - |

| Ward-level crime correlation for neighbourhoods used in this study (n=7, two SOAs from same ward). Underlined: p-value. Italicised: Correlation coefficient | | | | | | |
| --- | --- | --- | --- | --- | --- | --- |
|  | Violence, Sexual Crime and Robbery | Burglary | Theft | Criminal Damage | Drug-related | Other Crime |
| Violence, Sexual Crime and Robbery | - | 0.01 | <0.01 | <0.01 | <0.01 | <0.01 |
| Burglary | *0.89* | - | 0.01 | <0.01 | <0.01 | <0.01 |
| Theft | *1.00* | *0.88* | - | <0.01 | <0.01 | <0.01 |
| Criminal Damage | *0.97* | *0.97* | *0.96* | - | <0.01 | <0.01 |
| Drug-related | *0.99* | *0.93* | *0.98* | *0.99* | - | <0.01 |
| Other Crime | *1.00* | *0.91* | *0.99* | *0.98* | *0.99* | - |

| Ward-level correlations between crime and disorder scores, used as neighbourhood crime rate in this study, and individual crime rates for all wards of Northern Ireland (N=582). Underlined: p-value. Italicised: Correlation coefficient | | | | | | | | |
| --- | --- | --- | --- | --- | --- | --- | --- | --- |
|  | Crime and Disorder Score | All Crime | Violence, Sexual Crime and Robbery | Burglary | Theft | Criminal Damage | Drug-related | Other Crime |
| Crime and Disorder Score | - | <0.01 | <0.01 | <0.01 | <0.01 | <0.01 | <0.01 | <0.01 |
| All Crime | *0.62* | - | <0.01 | <0.01 | <0.01 | <0.01 | <0.01 | <0.01 |
| Violence, Sexual Crime and Robbery | *0.62* | *0.97* | - | <0.01 | <0.01 | <0.01 | <0.01 | <0.01 |
| Burglary | *0.53* | *0.78* | *0.69* | - | <0.01 | <0.01 | <0.01 | <0.01 |
| Theft | *0.47* | *0.94* | *0.87* | *0.67* | - | <0.01 | <0.01 | <0.01 |
| Criminal Damage | *0.72* | *0.94* | *0.91* | *0.79* | *0.81* | - | <0.01 | <0.01 |
| Drug-related | *0.58* | *0.89* | *0.87* | *0.74* | *0.79* | *0.83* | - | <0.01 |
| Other Crime | *0.6* | *0.93* | *0.91* | *0.65* | *0.86* | *0.86* | *0.82* | - |

**ESM 4.2: Correlation of Individual Perceptions of Neighbourhood Characteristics**

Correlations were tested between the perceptions of the median age at death (MAD), morbidity rate, crime rate and adult sex ratio.

In the tables below, correlation coefficients are italicised and p-values are underlined. Values of zero indicate where R has returned a value of zero as the actual value was negligible. P<0.05 indicated by ‘*’.

|  | MAD | Morbidity Rate | ASR | Safety |
| --- | --- | --- | --- | --- |
| MAD | - | 0.02* | 0.003 | 0.03* |
| Morbidity Rate | *-0.19* | - | 0.32 | 0.23 |
| ASR | *0.23* | *-0.08* | - | 0.09 |
| Safety | *0.17* | *-0.10* | *0.14* | - |
